# Single‐Cell Sequencing Redefines Immune Heterogeneity and Communication Networks in ARDS: Toward Precision Therapeutics

**DOI:** 10.1155/ijog/9902676

**Published:** 2025-10-21

**Authors:** Han-Bing Chen, An-Min Xu, Hai-Bo Qiu, Jie Chao, Songyun Zhao

**Affiliations:** ^1^ Jiangsu Provincial Key Laboratory of Critical Care Medicine, Department of Critical Care Medicine, Zhongda Hospital, School of Medicine, Southeast University, Nanjing, China, seu.edu.bd; ^2^ Jiangsu Provincial Key Laboratory of Critical Care Medicine, Department of Physiology, Key Laboratory of Environmental Medicine Engineering, Ministry of Education, School of Public Health, Southeast University, Nanjing, Jiangsu, China, seu.edu.bd; ^3^ Jiangsu Provincial Key Laboratory of Critical Care Medicine, Department of Physiology, School of Medicine, Southeast University, Nanjing, Jiangsu, China, seu.edu.bd

**Keywords:** ARDS, immune cell heterogeneity, intercellular communication, precision medicine, single-cell RNA sequencing

## Abstract

Acute respiratory distress syndrome (ARDS) is a critical condition characterized by diffuse alveolar damage and intense inflammatory responses. During the COVID‐19 pandemic, its incidence and mortality have remained persistently high. Conventional approaches have struggled to uncover the complex cellular heterogeneity and dynamic inflammatory networks underlying ARDS. The advent of single‐cell sequencing technologies has revolutionized our ability to dissect the molecular mechanisms of ARDS. This review systematically summarizes recent advances in the application of single‐cell sequencing in studying pulmonary inflammation in ARDS, with a focus on its strengths in elucidating immune cell heterogeneity, reconstructing intercellular communication networks, and identifying potential therapeutic targets. Furthermore, we discuss current technical limitations and translational challenges, aiming to provide a theoretical foundation and future direction for translating mechanistic insights into precision medicine for ARDS.

## 1. Introduction

Acute respiratory distress syndrome (ARDS) is a clinical syndrome caused by diffuse inflammatory injury to the alveoli and pulmonary capillaries, triggered by pulmonary or extrapulmonary insults. The hallmark pathological features of ARDS include disruption of the alveolar–capillary barrier, accumulation of protein‐rich pulmonary edema, and massive infiltration of inflammatory cells [1, 2]. Despite advances in respiratory support and treatment strategies, in‐hospital mortality remains as high as 30%–40% [3, 4] and is even higher in COVID‐19–related ARDS cases [5–7]. The poor prognosis of ARDS is largely attributable to its highly complex pathophysiology. Rather than a single disease entity, ARDS represents a common pathological endpoint resulting from diverse etiologies, involving widespread dysregulation of both innate and adaptive immune responses [8–11], disruption of epithelial and endothelial barriers [12–16], and aberrant activation of fibrotic repair pathways [17, 18].

Traditional methods for studying ARDS inflammation—such as histopathological analysis, flow cytometry, and bulk RNA sequencing (bulk RNA‐seq)—have laid the foundation for our current understanding of its pathology and immunology. However, these approaches have significant limitations. Histopathology provides static morphological information and fails to capture molecular dynamics; flow cytometry depends on predefined surface markers, making it difficult to identify novel or unexpected cell populations; and bulk RNA‐seq only measures average gene expression across mixed cell populations, masking inter‐ and intralineage heterogeneity. As a result, our understanding of ARDS pathogenesis has long remained at a coarse resolution, hindering the identification of precise disease‐driving mechanisms and therapeutic targets.

**Figure 1:**
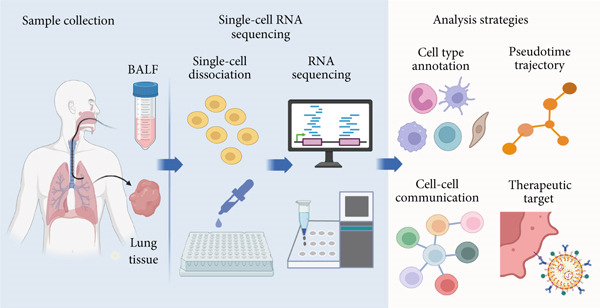
Overview of single‐cell sequencing workflow and analytical strategies in ARDS research. This graphical abstract summarizes the key steps in single‐cell RNA sequencing (scRNA‐seq) applied to ARDS, including sample acquisition (e.g., BALF and lung tissue), single‐cell dissociation, and transcriptomic profiling. Downstream analysis strategies—such as cell type annotation and pseudotime trajectory inference—enable the reconstruction of dynamic immune responses, intercellular communication networks, and identification of therapeutic targets. These approaches together facilitate a comprehensive understanding of ARDS immunopathology at single‐cell resolution.

The emergence of single‐cell sequencing has provided an unprecedented tool to dissect the complex cellular and molecular landscape of ARDS. This technology enables high‐resolution profiling of gene expression at the single‐cell level, facilitating the identification of rare subpopulations, transitional states, and functionally heterogeneous subsets that are often invisible to conventional techniques [19–21]. However, it is important to note that tissue dissociation required for single‐cell analysis may inadvertently result in the loss of fragile or rare populations, thereby introducing potential bias. This technical limitation should be carefully considered when interpreting findings [22, 23]. In ARDS studies, single‐cell RNA sequencing (scRNA‐seq) not only captures dynamic immune cell changes but also identifies pathogenic subsets, reconstructs intercellular communication networks, and uncovers potential therapeutic targets [24, 25] (Figure 1). Notably, during the COVID‐19 pandemic, scRNA‐seq has been extensively applied to virus‐associated ARDS, significantly enhancing our understanding of local immunopathological processes within the lung [26–28].

In recent years, single‐cell sequencing has evolved into a multimodal platform, integrating transcriptomic, proteomic, epigenetic, and immune receptor data. For example, CITE‐seq enables simultaneous detection of mRNA and surface proteins [29]; scATAC‐seq profiles chromatin accessibility [30, 31]; and scVDJ‐seq captures T/B cell receptor rearrangements [32, 33], allowing for a more comprehensive characterization of immune states. Meanwhile, the rise of spatial transcriptomic (ST) has addressed the lack of spatial context in conventional scRNA‐seq, enabling researchers to explore spatial heterogeneity in inflammatory responses while preserving tissue architecture [34–38].

## 2. Technical Workflow and Core Methodologies of scRNA‐seq in ARDS

A complete scRNA‐seq workflow typically involves five essential steps: sample preparation, single‐cell isolation, library construction, high‐throughput sequencing, and data analysis.

In ARDS‐related pulmonary inflammation research, common sample sources include bronchoalveolar lavage fluid (BALF) and lung tissue biopsies. Because most ARDS patients require mechanical ventilation, BALF is more accessible and is enriched in pulmonary immune cells, making it the most widely used sample type [39]. In contrast, lung tissue provides more direct histopathological information but is difficult to obtain and is usually sourced from autopsy or lung transplantation procedures.

Single‐cell isolation is a critical step for ensuring high‐quality data. Common isolation methods include fluorescence‐activated cell sorting (FACS) and droplet‐based microfluidic systems. Compared with FACS, droplet‐based platforms (e.g., 10x Genomics) offer higher throughput, lower cost, and greater operational simplicity, and have become the mainstream approach [40–42]. It is important to note that ARDS samples often contain abundant inflammatory cells and cellular debris. Therefore, sample pretreatment requires optimized centrifugation and lysis protocols to reduce cell loss and RNA degradation, thereby preserving single‐cell integrity. Several strategies can improve cell viability during sample preparation, such as minimizing processing time, using gentle enzymatic digestion combined with mechanical dissociation, maintaining samples at low temperature to reduce stress responses, and supplementing buffers with viability‐enhancing agents (e.g., ROCK inhibitors or antioxidants) [43, 44].

During the library construction stage, reverse transcription and cDNA amplification steps may introduce technical biases, and such biases vary across platforms (Table 1). Full‐length transcript sequencing platforms like SMART‐seq2 are suitable for low‐throughput but high‐sensitivity studies [53, 54], while 3 ^′^‐end sequencing platforms like 10x Genomics are more appropriate for large‐scale cellular atlases. Given the widespread transcriptional changes in immune pathways under inflammatory conditions, 3 ^′^‐end sequencing is generally sufficient for most ARDS studies.

**Table 1:** Representative immune cell subsets identified by scRNA‐seq in ARDS.

**Immune lineage**	**Subset (scRNA-seq label)**	**Key functional roles**	**Involved pathways**	**Representative studies**
Macrophages	Monocyte‐derived macrophages	Activate fibrotic remodeling; suppress antigen presentation	TGF‐*β*/SMAD, CCR2 axis	[45]
	Slamf9^+^ macrophages	Dual‐phase function: antiviral → prorepair	IFN response, inflammation resolution	[46]
	Ly6G^+^ hybrid macrophages	Bridge monocyte–neutrophil phenotypes; amplify inflammation	CCR2^+^ trafficking	[47]

Neutrophils	Fth1^high^ neutrophils	Sustain inflammation, impair resolution	IL‐10‐dependent loop, NETosis	[48]
	Prok2^high^ neutrophils	Mediate early antimicrobial response, tissue repair	Chemokine burst	
	Reverse‐migrated neutrophils (rTEM)	Re‐enter circulation; propagate systemic inflammation	Endothelial EVs, IL‐6/STAT3	[49]

CD8^+^ T cells	Exhausted TRM‐like CD8^+^ T cells	Impaired cytotoxicity and clonal expansion	PD‐1/CCR5/CXCL11 axis	[50]

B cells	IGHV1‐18^+^ IGLV3‐20^+^ plasmablasts	Undergo SHM but limited lung infiltration	Germinal center genes, SHM machinery	[51]

Others	MX1^+^ NK cells, activated DCs, ILCs	Modulate macrophage activity; orchestrate immune crosstalk	IFN/IL‐12 signaling, CCR7 axis	[52]

Data analysis is the core process for extracting biological insights from single‐cell data. Standard pipelines typically involve quality control, normalization, dimensionality reduction, clustering, and cell type annotation. ARDS datasets often exhibit both technical (e.g., intersample variation in cell composition) and biological (e.g., rapid inflammatory fluctuations) heterogeneity, which necessitates more adaptive computational strategies. For example, batch correction algorithms such as Harmony and BBKNN can be used to integrate timepoint‐specific or multicenter datasets. Furthermore, since immune cells in ARDS undergo continuous activation and differentiation, pseudotime analysis and RNA velocity approaches are often more suitable than traditional static clustering methods for capturing dynamic trajectories [55–58].

Beyond the standard workflow of scRNA‐seq, a range of advanced analytical approaches have proven particularly informative for dissecting ARDS and its immune complexity. Trajectory inference and RNA velocity methods (e.g., Monocle, Slingshot, and scVelo) enable reconstruction of dynamic differentiation pathways, such as the transition from circulating monocytes to proinflammatory or profibrotic macrophages, as well as the temporal evolution of neutrophil states during acute injury and resolution [57, 59, 60]. Ligand–receptor inference tools (CellChat, CellPhoneDB, and NATMI) are widely used to map intercellular communication networks, revealing how chemokine‐ and cytokine‐driven loops between macrophages, neutrophils, T cells, and stromal cells amplify inflammatory cascades in ARDS [61, 62]. Integration with ST adds critical context, allowing transcriptional states to be mapped back to tissue architecture and uncovering spatially organized immune–stromal interactions within injured alveoli [63]. Finally, multiomics integration strategies that combine scRNA‐seq with ATAC‐seq, proteomic, or genome‐wide association studies (GWAS) datasets are increasingly applied to link epigenetic regulation and host susceptibility with cellular phenotypes [60, 63]. Together, these approaches move beyond static cataloguing to provide mechanistic insights into immune heterogeneity, highlighting context‐specific and potentially targetable pathways in ARDS.

## 3. Single‐Cell Atlases Reveal Immune Cell Heterogeneity and Dynamic Evolution in ARDS

In the early stages of ARDS research, bulk RNA‐seq was commonly employed, which only captured average transcriptional signals at the population level. This limitation contributed to a simplified understanding of the immune landscape. The emergence of single‐cell sequencing has overcome these constraints, revealing the continuum and complexity of immune cell states, leading to the discovery of novel pathogenic subpopulations, and fundamentally reshaping our understanding of ARDS immunopathology.

### 3.1. Immune heterogeneity in ARDS

Immune dysregulation is a hallmark of ARDS, characterized by complex interactions among innate and adaptive immune populations in the injured lung. Earlier approaches have provided important but often partial insights into this complexity. Bulk transcriptomic studies of lung tissue and peripheral blood revealed overarching signatures of hyperinflammation, interferon‐driven responses, and compensatory immunosuppression [64], but the averaging of cell populations inevitably obscured cell‐type–specific contributions. Flow cytometry extended this knowledge by identifying quantitative changes in circulating monocytes, neutrophils, and T cell subsets associated with disease severity [65]. CyTOF (mass cytometry) further extended resolution by enabling simultaneous measurement of dozens of surface and intracellular markers, thereby uncovering greater phenotypic diversity and rare immune subsets not captured by conventional flow cytometry. Nevertheless, both methods are constrained by antibody panel design and limited in capturing novel or unexpected cell states [66]. More recently, spatial profiling approaches have begun to reveal how inflammatory immune cells cluster with stromal and epithelial compartments in damaged alveolar regions, highlighting spatially organized immune–stromal interactions [50, 51], though resolution remains coarser than at the single‐cell level. Against this backdrop, single‐cell sequencing refines and expands prior frameworks by providing unbiased, genome‐wide views of immune cell states at unprecedented resolution. These approaches have uncovered novel pathogenic subsets. Moreover, single‐cell data permit inference of intercellular communication networks, revealing cross‐talk between myeloid and lymphoid populations, as well as stromal remodeling programs linked to fibrotic progression and outcomes. Thus, when viewed in context, single‐cell studies not only corroborate earlier findings from bulk and cytometric approaches but also fundamentally extend them, redefining the landscape of immune heterogeneity in ARDS.

### 3.2. Heterogeneity and Dynamic Trajectories of Macrophages and Neutrophils

Macrophages are central regulators of pulmonary inflammation in ARDS [67]. scRNA‐seq studies have shown that their phenotypic and functional diversity far exceeds the classical M1/M2 polarization model. In a mouse model of influenza‐induced lung injury, Cecilia et al. identified a novel Ly6G^+^ macrophage subpopulation originating from bone marrow progenitors. These cells migrated to inflamed tissues via a CCR2‐dependent pathway and exhibited hybrid features of neutrophils and conventional macrophages, reflecting blurred lineage boundaries and high plasticity [47].

In COVID‐19–associated ARDS, monocyte‐derived macrophages are markedly enriched in BALF and display a damage‐associated phenotype. The group led by Leif Erik Sander demonstrated that these macrophages not only respond to tissue injury but also activate profibrotic pathways such as transforming growth factor (TGF)‐*β*/SMAD signaling proteins. Spatial colocalization with fibroblasts and upregulation of matrix remodeling genes suggest that these immune cells may initiate early fibrotic processes even before the resolution of acute infection, thereby increasing the risk of long‐term pulmonary fibrosis [45]. Similarly, in the same context, Cong et al. identified a Slamf9^+^ macrophage subset with dual‐phase functionality: exhibiting antiviral activity during the acute phase and promoting inflammation resolution during recovery [46]. These findings challenge the static activation paradigm and support the concept of dynamic reprogramming within the pulmonary mononuclear phagocyte system. In sepsis‐associated ARDS, scRNA‐seq of PBMCs delineated distinct monocyte subclusters, revealing a SOCS3^low^ transcriptional program with heightened Type I IFN responsiveness, particularly in CD16^+^ cells. This subset‐level divergence was coupled with upregulation of genes such as RAB11A, ATP2B1, and SPARC, highlighting how differential monocyte states contribute to sustained neutrophil inflammation, endothelial dysfunction, and fibroproliferation [68].

Neutrophils, traditionally viewed as terminal effector cells, also display diverse transcriptional and functional states in ARDS. In an LPS‐induced lung injury model, scRNA‐seq identified two distinct neutrophil subtypes: Fth1^high^ and Prok2^high^. The former contributed to sustained inflammation and tissue damage, while the latter was involved in early defense and tissue repair [48]. This suggests that neutrophils can switch between protective and pathogenic roles depending on temporal and environmental cues.

Another notable discovery is the phenomenon of reverse transendothelial migration (rTEM) neutrophils. In sepsis‐associated ARDS, these cells can re‐enter the bloodstream after traversing pulmonary tissue, potentially amplifying systemic inflammation and contributing to remote organ dysfunction [49]. Increasing evidence also suggests that neutrophils can participate in antigen presentation and engage in complex crosstalk with adaptive immune cells [69]—a function previously underappreciated. The integration of scRNA‐seq with trajectory analysis can help elucidate whether neutrophils promote or resolve inflammation across different ARDS phenotypes and assess their potential as therapeutic targets, particularly for identifying profibrotic or hyperreactive subsets.

### 3.3. Lymphocyte Remodeling and Aberrant Differentiation Trajectories

Lymphocytes act as both regulators and executors of immune responses in ARDS. Dysregulation in their function or differentiation is a key contributor to pathological inflammation and impaired tissue repair. High‐resolution single‐nucleus RNA‐seq (snRNA‐seq) analyses of lung tissues from fatal COVID‐19 cases have revealed profound lymphoid lineage remodeling, characterized by disruption of effector differentiation pathways. T cells and natural killer (NK) cells commonly exhibited downregulation of tissue‐resident memory markers and cytotoxic programs (e.g., GZMB), along with a lack of compensatory clonal expansion—suggesting an “exhaustion‐like” state with arrested differentiation.

Although certain plasmablast subsets (e.g., IGHV1‐18–IGLV3‐20) displayed high somatic hypermutation (SHM) and clonal expansion, overall B cell infiltration in the lung remained limited, implying that humoral immunity alone may be insufficient to compensate for T cell dysfunction [51]. ST analysis further revealed “compartmentalized disorganization” of lymphocytes within the lung—lymphocytes were scarce and functionally impaired in myeloid‐dominant regions, whereas mixed immune regions exhibited concurrent activation and exhaustion states. This suggests that local microenvironmental heterogeneity profoundly shapes lymphocyte fate. It should be noted that most current ST platforms, such as 10x Visium and Slide‐seq, profile gene expression at the spot level, where each spot typically contains multiple cells rather than providing true single‐cell resolution. Nevertheless, when integrated with scRNA‐seq datasets, ST has proven powerful in mapping transcriptional states back to their tissue context and in uncovering spatially organized immune circuits within injured lungs [70–72].

In severe COVID‐19–related ARDS, paradoxical coexistence of T/NK cell activation and exhaustion phenotypes is particularly prominent. Integrated analyses combining scRNA‐seq with GWAS revealed that exhaustion signatures in CD8^+^ T cells are significantly enriched in severe cases, with upregulation of CCR5 colocalizing with chemokine gradients (e.g., CXCL11) in inflamed alveolar regions [50]. These findings imply that host genetic susceptibility may exacerbate local immune exhaustion and functional collapse.

Most studies of lymphocyte heterogeneity in ARDS to date have focused on COVID‐19 populations and rely primarily on peripheral blood mononuclear cells (PBMCs), which reflect systemic immune states but fail to fully capture the dynamics of lung‐resident lymphocytes [73]. In contrast, direct analyses of BALF or lung tissue in non–COVID‐19 ARDS—particularly regarding T cell development, exhaustion, and clonal expansion—remain limited. Future studies should compare T cell trajectories across infectious and noninfectious ARDS etiologies, integrating pseudotime analysis and T cell receptor sequencing to identify common or distinct immune checkpoints, which could serve as subtype‐specific therapeutic targets.

### 3.4. Understudied Immune Lineages and Future Opportunities

Beyond T and B cells, other immune lineages—including dendritic cells (DCs), innate lymphoid cells (ILCs), and NK cells—are increasingly recognized as critical yet undercharacterized players in ARDS pathogenesis [74–78]. DCs play central roles in antigen presentation and T cell priming [79, 80]; however, their plasticity and subset‐specific functions in ARDS‐associated lung inflammation remain poorly defined. Single‐cell profiling may reveal functional distinctions among DC subsets in mediating immune tolerance versus activation [52, 81]. Although NK cells are traditionally known for cytotoxic functions, emerging evidence suggests they also participate in immunoregulation by influencing macrophage activity, modulating T cell responses, and even suppressing fibrosis [82–86]. Their precise role in ARDS warrants further investigation. Systematically incorporating these “nonmainstream” lineages into future single‐cell studies—especially in combination with spatial and temporal analyses—will be a key to building a comprehensive immune atlas of ARDS and evaluating the translational potential of these cell types.

### 3.5. Integrative Mechanistic Perspective on Immune Heterogeneity in ARDS

**Figure 2:**
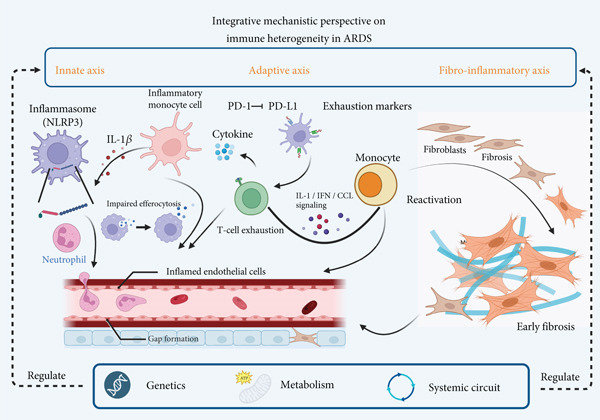
Integrative mechanistic perspective on immune heterogeneity in ARDS. This schematic integrates innate, adaptive, and fibro‐inflammatory axes of ARDS immunopathology. It highlights NLRP3 inflammasome activation and impaired efferocytosis by macrophages, excessive neutrophil activation and NET formation, T cell exhaustion via PD‐1/PD‐L1 signaling, and macrophage–fibroblast crosstalk driving fibrosis through IL‐1, IFN, and CCL pathways.

Beyond descriptive cataloguing, accumulating evidence suggests that immune heterogeneity in ARDS can be distilled into several convergent mechanistic axes that shape disease trajectory. First, innate immune dysregulation involves inflammasome activation, impaired efferocytosis, and neutrophil extracellular trap (NET) accumulation, collectively disrupting the alveolar–capillary barrier [87, 88]. Second, adaptive immune dysfunction emerges through exhausted T and NK cell states, marked by impaired cytotoxicity and clonal expansion, compromising pathogen clearance while perpetuating inflammation [89]. Third, fibroinflammatory remodeling arises from maladaptive macrophage–fibroblast crosstalk, particularly via TGF‐*β*/SMAD signaling, linking acute inflammation to early fibrosis and long‐term lung dysfunction [90, 91]. These axes are further modulated by genetic susceptibility, metabolic rewiring, and systemic immune circuits, underscoring that heterogeneity is not merely phenotypic but mechanistically embedded. As summarized in Figure 2, this integrative framework highlights how diverse immune trajectories converge into distinct ARDS endotypes, offering a conceptual basis for precision therapeutic strategies.

### 3.6. Annotation Heterogeneity Across Studies

A recurring challenge in the interpretation of scRNA‐seq studies on ARDS lies in the lack of standardized annotation frameworks, which leads to divergent definitions of disease‐associated immune populations. These discrepancies are not merely semantic but can shift the biological interpretation of ARDS pathogenesis.

Macrophages exemplify this issue most prominently. Clusters with overlapping transcriptional profiles—such as CD14^+^HLA‐DR^low^ monocyte‐derived macrophages—have been variously labeled as inflammatory, damage‐associated [57, 92, 93], or profibrotic subsets enriched for TGF‐*β* signaling. Each label emphasizes a different pathogenic axis (acute inflammation, tissue injury, or maladaptive remodeling), complicating cross‐study synthesis [45, 94]. Neutrophils are another case in point. Some reports highlight oxidative stress–dominant subsets in bacterial or sterile ARDS models, whereas others emphasize interferon‐stimulated or chemokine‐enriched neutrophils in viral ARDS, particularly COVID‐19 [95, 96]. These divergent classifications reflect both the plasticity of neutrophil responses and methodological differences in clustering and marker selection. T and NK cells similarly suffer from inconsistent annotation. Exhausted CD8^+^ T cells have been variably described as dysfunctional, exhausted‐like, or arrested differentiation states, depending on the analytical framework. Likewise, NK cells have been characterized as either cytotoxic effectors or immunoregulatory subsets, with different studies emphasizing activation, suppression, or tissue residency features [97–99].

Together, these inconsistencies highlight the need for harmonized nomenclature and reference frameworks in ARDS single‐cell research. While some of the observed heterogeneity likely reflects genuine context‐specific biology—driven by etiology, sampling site, or disease stage—other differences arise from analytical choices and annotation practices. Future efforts should prioritize consensus‐building and integrative cross‐study comparisons to distinguish universally conserved disease‐driving populations from context‐dependent phenomena.

## 4. Cross‐Etiological Perspectives: Integrating Non–COVID‐19 ARDS Studies

While the majority of available single‐cell datasets are derived from COVID‐19–related ARDS, our review intentionally integrates all etiologies studied to date. Non–COVID‐19 ARDS investigations—including sepsis‐, aspiration‐, chemical injury–, and LPS‐induced models—are fewer in number and generally involve smaller cohorts, yet they provide crucial complementary perspectives. Comparative analysis across these etiologies reveals both shared and distinct features: Myeloid‐driven inflammation and neutrophil plasticity appear to be conserved hallmarks across ARDS, whereas sepsis is marked by an immunoregulatory skew exemplified by SOCS3^low^ monocytes, and chemical injury highlights unique epithelial trajectories such as transitional AT2‐to‐AT1 states. By contrast, aspiration and LPS‐induced ARDS are characterized by more acute neutrophilic influx and injury‐associated inflammatory circuits. To improve transparency, we summarize all non–COVID datasets, including their sample sizes and sources, in Table S1 [48, 49, 68, 89, 100–110]. Together, these findings underscore that ARDS immune heterogeneity is not a phenomenon unique to viral ARDS, but rather spans a spectrum of etiologies, with both conserved mechanisms and context‐specific trajectories. Future cross‐etiological, longitudinal studies will be essential to disentangle shared pathways from etiology‐dependent programs, thereby improving the generalizability of single‐cell insights.

## 5. Unraveling Inflammatory Communication Networks in ARDS Through Single‐Cell Analysis

### 5.1. Reconstructing Cell–Cell Interaction Networks via Ligand–Receptor Analysis

Hyperactivation of inflammatory signaling pathways and dysregulated intercellular communication are widely recognized as central features of ARDS pathogenesis. The advent of scRNA‐seq has enabled researchers to reconstruct intercellular interaction networks at a systems level, offering unprecedented resolution to unravel the complexity of immune communication. By integrating single‐cell transcriptomic data with curated ligand–receptor interaction databases—such as CellPhoneDB, CellChat, and NATMI—computational frameworks can reconstruct communication networks between immune and stromal cells within the inflamed pulmonary microenvironment (Table 2).

**Table 2:** Representative immune cell subsets identified by scRNA‐seq in ARDS.

**Tool**	**Core algorithm/output**	**Supports directionality**	**Species**	**Database coverage**	**Database comprehensiveness**	**Representative reference**
CellPhoneDB	Pairwise coexpression inference	No	Human, mouse	Curated LR pairs, multi‐subunit	High; frequently curated, widely adopted	[111]
CellChat	Probabilistic modeling of signaling pathways	Partial	Human, mouse	CellChatDB; pathway‐level curated	High; pathway‐ integrated.regularly updated	[112]
NATMI	Network‐based interaction scoring	Yes	Multispecies	ConnectomeDB	Moderate‐high; broad but slower updates	[113]
NicheNet	Ligand activity inference via gene regulation	Yes	Human, mouse	LRBase + regulatory networks	High; functional downstream effects, less frequent updates	[114]

In COVID‐19–associated ARDS, this approach has been widely applied to BALF samples. Liao et al. demonstrated that alveolar macrophages are major sources of inflammatory mediators such as interleukin (IL)‐1*β* and CXCL8, and that these signals are negatively correlated with T cell functionality, suggesting a macrophage‐driven negative feedback loop that may exacerbate lymphocyte exhaustion and immune imbalance [92]. Wauters et al. further revealed that, in severe COVID‐19 patients, communication between macrophages and T cells via IL‐1, interferon, and CCL pathways is significantly enhanced [57], underscoring the pathological crosstalk between innate and adaptive immunity in driving lung injury. Beyond BALF, Delorey et al. analyzed lung tissue from deceased COVID‐19 patients and identified a prominent TGF‐*β*– and IL‐1–mediated communication axis between macrophages and fibroblasts, accompanied by coexpression of extracellular matrix remodeling genes and fibrotic markers [50, 51]. These findings suggest that in some patients, fibrotic pathways may be aberrantly activated during the acute inflammatory phase, predisposing them to long‐term pulmonary fibrosis.

Although most current studies have focused on virus‐induced ARDS, similar analytical frameworks are equally applicable to bacterial and noninfectious ARDS models. In the future, integrating intercellular communication atlases across different ARDS etiologies—including viral, bacterial, chemical, and traumatic—will be crucial for identifying conserved inflammatory circuits and targetable disease‐specific vulnerabilities.

### 5.2. From Transcript to Function: Advances in Predicting and Validating Inflammatory Signaling

Despite the widespread use of ligand–receptor analysis in ARDS research, most current strategies face inherent limitations in biological interpretability. One major constraint is the reliance on mRNA expression to infer signaling events, which does not always reflect protein abundance or activity. Moreover, traditional prediction models often fail to distinguish between paracrine and autocrine signaling and typically overlook the impact of spatial tissue organization on communication dynamics [115, 116]. To overcome these limitations, more sophisticated computational tools are being developed. For example, NicheNet integrates ligand–receptor expression with downstream gene expression programs in target cells, enabling inference of the “functional consequences” of predicted interactions. This approach not only identifies potential ligands but also predicts whether they drive key transcriptional responses in recipient cells [114].

A further frontier in the field is the integration of ST with single‐cell communication models. By localizing ligand and receptor expression within specific anatomical regions, spatial data can reveal whether certain cell–cell interactions are confined to particular lung microenvironments—such as peribronchial spaces, alveolar lumens, or fibrotic foci [117, 118]. This is especially important in ARDS, where regional heterogeneity in cellular composition and microenvironmental cues may significantly shape the occurrence and relevance of specific signaling events. Early studies using the 10x Genomics Visium platform have integrated ST data with scRNA‐seq to identify communication “hotspots” in COVID‐19 lungs—such as the colocalization of TGF‐*β*–expressing macrophages with ACTA2^+^ fibroblasts [119]. These observations suggest that the physical proximity of immune and structural cells is critical for effective pathological signal transmission, which cannot be discerned by scRNA‐seq alone. Collectively, the integration of scRNA‐seq, ST, and functional regulatory networks is ushering in a new phase of ARDS research—one that moves beyond speculative interactions toward mechanistic validation and targeted intervention.

### 5.3. Extracellular Vesicles (EVs) as Emerging Mediators of Intercellular Communication in ARDS

In addition to classical ligand–receptor interactions, EVs—including exosomes and microvesicles—have recently emerged as important but underrecognized mediators of intercellular communication in ARDS. EVs can transport diverse molecular cargo such as mRNAs, miRNAs, lipids, and proteins and can modulate the behavior of target cells either locally or at distant sites [25, 49, 120–123].

Although traditional EV research has relied on proteomics or nanoparticle tracking analysis, emerging single‐cell studies have begun to infer EV‐related functions by examining the expression of EV biogenesis and trafficking genes. Genes such as CD63, TSG101, and ALIX—key components of exosome formation—and members of the RAB GTPase family involved in vesicle trafficking, show cell‐type–specific expression patterns in ARDS lung tissues, including in macrophages, neutrophils, and epithelial cells [124]. One study reported that macrophage‐derived EVs can induce necroptosis and inflammatory responses in epithelial cells in vitro, suggesting that EVs not only regulate immune responses but may also directly contribute to tissue injury [125]. Importantly, EVs carry not only protein cargo but also noncoding RNAs, including miRNAs and long noncoding RNAs (lncRNAs), which have been shown to play critical roles in modulating immune responses in ARDS [126, 127]. The integration of scRNA‐seq, EV omics, and ST may eventually reveal a cross‐cell, cross‐region RNA signaling network within ARDS lungs. Although still in its early stages, research on EV‐mediated communication offers a novel perspective on immune regulation and tissue injury, particularly in contexts where traditional ligand–receptor models fail to explain long‐range or indirect signaling events.

## 6. Cross‐Tissue and Systemic Features of Immune Remodeling in ARDS

Although ARDS is traditionally defined as a severe pulmonary inflammatory syndrome, emerging evidence from single‐cell studies has revealed that its immunopathological processes extend far beyond the lungs. Rather than being confined to a single organ, ARDS involves immune alterations across the lungs, blood, and even distal organs, forming a coordinated and systemic inflammatory network that may contribute to the development of multiple organ dysfunction syndrome (MODS).

In the peripheral blood, scRNA‐seq studies have identified significant changes in both myeloid and lymphoid compartments, such as the expansion of CD14^+^HLA‐DR^low^ monocytes and the emergence of dysfunctional T cell subsets. These changes are not isolated events, as corresponding immune programs—such as macrophage polarization, neutrophil activation, and impaired antigen presentation—have also been observed in the lungs. Notably, certain immune features, including Type I interferon responses and profibrotic transcriptional programs, appear to be shared between pulmonary and systemic compartments, suggesting the presence of cross‐tissue inflammatory axes [73].

Furthermore, advances in TCR and BCR clonotype analyses have revealed the presence of shared lymphocyte clones between lung tissue and peripheral blood. This clonal convergence implies the existence of systemic antigenic drivers as well as potential recirculation or trafficking of adaptive immune cells. These shared T cell clones are often enriched in exhaustion or cytotoxicity‐related gene signatures, reinforcing the notion of a unified cross‐compartment immune response [128].

Emerging evidence suggests that the immune dysregulation observed in ARDS may not be strictly confined to the lung, particularly in the context of sepsis‐induced systemic inflammation. Although most mechanistic insights are derived from pulmonary tissue, some clinical models of sepsis have reported extrapulmonary immune perturbations. For instance, systemic inflammatory responses can lead to Kupffer cell activation in the liver and elevated IL‐6 production, potentially engaging STAT3‐mediated acute‐phase responses [129]. Similarly, renal inflammation, including chemokine‐driven monocyte infiltration and oxidative stress, has been described in septic models [130]. While these findings do not specifically define ARDS pathophysiology, they underscore the plausibility of interorgan immune cross‐talk and the need for further investigation into systemic immunopathology in ARDS.

Taken together, ARDS‐associated immune imbalance should be conceptualized as a multiorgan, systemic process. While the lung remains the principal site of damage, its inflammatory signals may propagate systemically through mechanisms such as immune cell migration, cytokine diffusion, and neuroimmune crosstalk. Future studies should aim to characterize the composition and dynamics of immune populations in extrapulmonary organs and integrate immune profiling from BALF, lung tissue, peripheral blood, and distal organs to establish a multidimensional and spatially resolved immune monitoring framework. Such efforts will be critical to advancing a comprehensive understanding of ARDS pathogenesis and informing precision therapeutic strategies.

## 7. Translational Challenges of Single‐Cell Discoveries in ARDS

Single‐cell sequencing has provided a wealth of immunological insights and potential therapeutic targets in ARDS. However, significant barriers remain in translating these findings into clinical practice. These challenges span experimental validation, computational analysis, and clinical application, reflecting both the inherent complexity of ARDS and the current limitations of available technologies.

### 7.1. Functional Validation Bottlenecks

A major hurdle in the translational pathway is the lack of suitable platforms for functional validation. Most scRNA‐seq findings are correlational and do not establish causality. Traditional animal models such as LPS‐ or acid‐induced murine ARDS only partially mimic human disease and often fail to capture its cellular and molecular complexity [131]. Furthermore, several cell subsets identified in human single‐cell datasets have no clear analogs in murine systems or behave differently in rodents [132]. This species‐specific discrepancy limits the extrapolation of results and complicates preclinical drug testing.

To address this, recent efforts have turned toward more human‐relevant models. Cocultures of alveolar organoids with immune cells allow the reconstruction of epithelial–immune interactions. Precision‐cut lung slices (PCLS) preserve the three‐dimensional lung architecture, including alveolar, vascular, and immune niches, and support ex vivo infection or drug testing. Air–liquid interface cultures derived from patient samples also provide a controllable system to test inflammatory responses and cell–cell signaling [133–136]. Additionally, humanized mouse models—such as NOD‐scid IL2R*γ* null mice engrafted with human hematopoietic stem cells—offer in vivo systems to study human immune dynamics [137, 138]. Although promising, these models are limited by cost, scalability, and technical complexity, making them difficult to integrate into large‐scale screening pipelines.

ST and multiplexed imaging tools (e.g., CODEX, MIBI, and MERFISH) are emerging as valuable techniques for in situ validation of predicted cellular interactions. These approaches can retain spatial context and cellular topography, enabling researchers to confirm the proximity and directionality of ligand–receptor interactions predicted by scRNA‐seq [139–142]. However, these tools remain technically demanding and are not yet widely applied in ARDS research.

### 7.2. Technical and Computational Limitations

Beyond biological modeling, there are substantial technical and computational limitations that impact data reliability and interpretability. Droplet‐based platforms like 10x Genomics tend to underrepresent large or fragile cells such as megakaryocytes or alveolar macrophages, leading to biased cell‐type proportions [143]. Most protocols rely on 3 ^′^‐end sequencing, which limits isoform analysis and omits many noncoding RNAs that may regulate immune responses [144, 145]. Inflammatory conditions such as ARDS may involve significant contributions from circular RNAs, lncRNAs, or microbial transcripts—all of which are largely missed in current pipelines [146, 147].

Batch effects and inconsistent cell annotations further hinder cross‐study comparison. Differences in sample processing, sequencing depth, reference genomes, and clustering algorithms often lead to divergent or irreproducible subpopulation annotations. For example, the same cluster may be labeled as “inflammatory macrophages” in one study and “monocyte‐derived macrophages” in another. While computational tools such as Harmony, LIGER, and Seurat’s integration modules mitigate some of these issues, annotation consistency across studies remains below 70%, especially for highly plastic or transitional cell states [148]. This inconsistency complicates efforts to identify reproducible therapeutic targets or biomarkers.

In addition, most single‐cell pipelines focus on transcriptomic features without integrating other dimensions of cell identity such as surface protein expression, chromatin accessibility, or metabolic state. Multiomics approaches like CITE‐seq, scATAC‐seq, and spatial metabolomics hold promise for a more comprehensive characterization but remain underutilized in ARDS.

### 7.3. Biological and Clinical Translation Barriers

Even when biological insights are robust, translating them into actionable clinical strategies is nontrivial. Candidate targets must demonstrate not only mechanistic relevance but also therapeutic tractability and safety. Many promising targets are pleiotropic and may play context‐specific roles in extrapulmonary organs like the liver, kidney, or heart. This raises concerns about off‐target effects and necessitates more precise delivery strategies or context‐aware intervention timing [149–152].

Peripheral and lung immune landscapes differ substantially, and many critical cell populations in the lung are absent in PBMC samples [153]. This discrepancy complicates the use of blood‐based biomarkers and underscores the importance of accessing airway or lung tissue compartments for immunomonitoring. Moreover, most scRNA‐seq–derived targets lack functional validation in preclinical models or clinical settings. Despite identifying dysregulated pathways or unique cell subsets, few studies have integrated these findings into drug development pipelines or designed interventional trials based on molecular subtyping.

Another major obstacle is the limited ability to longitudinally monitor immune changes during ARDS progression. Most studies rely on cross‐sectional sampling, which fails to capture the dynamic nature of immune responses. Serial sampling of BALF or minimally invasive airway secretions, combined with rapid sequencing platforms, could enable time‐resolved tracking of disease states and response to interventions.

## 8. Future Perspectives: Advancing Single‐Cell–Guided Precision Medicine in ARDS

To overcome these challenges and realize the full translational potential of single‐cell insights, several key future directions must be prioritized. These efforts should aim to bridge the gap between descriptive cellular atlases and actionable clinical tools that inform prognosis, guide therapy, and stratify patients for tailored interventions.

### 8.1. Constructing Reference Atlases Across ARDS Etiologies

A foundational step is the development of large‐scale, standardized single‐cell reference atlases for ARDS. Most current studies focus on COVID‐19–related ARDS, with heterogeneous data quality, incomplete cellular coverage, and variable metadata annotation. Systematic collection of BALF, lung tissue, and PBMCs across diverse ARDS etiologies (e.g., trauma, bacterial infection, aspiration, and pancreatitis) and disease stages (early, late, and resolution) is essential. These efforts should adhere to common sample processing protocols, sequencing depth benchmarks, and annotation standards.

International initiatives such as the Human Lung Cell Atlas and the Lung MAP consortium should be leveraged to build cross‐platform, longitudinal single‐cell databases that capture immune dynamics over time. By integrating multiomic and clinical metadata, such atlases will support reproducible comparisons, cross‐cohort validation, and machine learning–based prediction models. These resources could also identify conserved or etiology‐specific cell states, facilitating the development of universal vs. targeted interventions [154, 155].

### 8.2. Developing Single‐Cell–Based Clinical Subtyping Systems

There is an urgent need to integrate high‐resolution immunological features into ARDS clinical stratification frameworks. Existing subtypes—such as hyperinflammatory versus hypoinflammatory—are largely based on peripheral cytokines or clinical scores and lack cellular granularity [156, 157]. Future models should incorporate signatures such as T/NK cell exhaustion, profibrotic macrophage states, aberrant antigen presentation, or dysregulated communication axes [158].

These features could be distilled into composite immune scores and linked with clinical metadata, including ventilator‐free days, steroid response, or secondary infection risk. Machine learning algorithms may help develop probabilistic subtyping models that assign patients to specific immune trajectories based on a limited set of blood or airway markers. Integration with electronic health records (EHRs) and point‐of‐care diagnostics could further support real‐time classification and therapeutic decision‐making.

### 8.3. Building Closed‐Loop Validation and Therapeutic Pipelines

**Figure 3:**
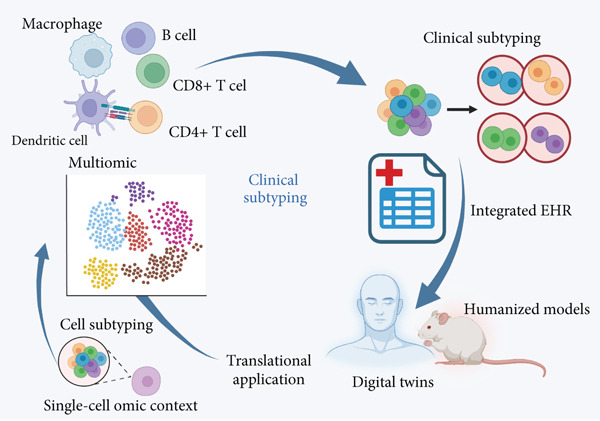
A conceptual framework for single‐cell–guided precision medicine in ARDS. This diagram illustrates a theoretical framework for implementing precision medicine in ARDS based on single‐cell sequencing. It encompasses three core components: reference atlas construction, clinical subtyping, and translational applications. The framework integrates multiomic single‐cell data, standardized annotation, and EHRs to enable the development of digital twin models and humanized systems for functional validation and therapeutic prediction.

A comprehensive bench‐to‐bedside pipeline must be established to translate scRNA‐seq findings into interventions (Figure 3). Candidate pathways should be tested in humanized systems, organoids, and ex vivo platforms such as PCLS. High‐throughput drug screening using patient‐derived cell models could accelerate discovery of context‐specific modulators [159].

Biomarker‐guided clinical trials should evaluate stratified treatment efficacy in molecularly defined ARDS subgroups. Rather than one‐size‐fits‐all approaches, future trials may enroll patients based on the presence of specific immune phenotypes, such as interferon‐driven inflammation, fibrotic macrophage expansion, or impaired antigen presentation. Adaptive trial designs could help refine inclusion criteria and response predictors over time.

Integration of scRNA‐seq with proteomics, cytokine profiling, metabolomics, and epigenetics may enable the creation of digital twin models—computational avatars of individual patients that simulate personalized disease trajectories and therapeutic responses. These models could support in silico trial design and virtual intervention testing.

Technological innovations such as portable sequencing devices, automated sample processing, and cloud‐based analytics will be key to bridging the final gap between discovery and bedside application. With continued interdisciplinary efforts, single‐cell data may soon support precision immunotherapy in ARDS at both the population and individual levels.

## 9. Conclusion

scRNA‐seq has fundamentally transformed our understanding of ARDS by enabling high‐resolution dissection of immune heterogeneity, intercellular signaling, and pathogenic cell states. Moving beyond descriptive frameworks, scRNA‐seq provides a mechanistic lens through which ARDS can be redefined not as a single entity, but as a spectrum of immunological endotypes. The integration of ST, multiomics profiling, and translational modeling is paving the way for precision medicine strategies tailored to immune dynamics at the single‐cell level. As this field advances, bridging the gap between single‐cell discoveries and therapeutic interventions will be critical for initiating a new era of individualized ARDS care.

## Ethics Statement

The authors have nothing to report.

## Disclosure

All authors have read and approved the manuscript.

## Conflicts of Interest

The authors declare no conflicts of interest.

## Author Contributions

H‐B.C. and A‐M.X. contributed equally to this work. H‐B.C. conceived of and designed the study. A‐M.X. performed analysis and generated the figures. H‐B.C. and A‐M.X. wrote the manuscript. H‐B.Q. and J.C. critically reviewed the manuscript. H‐B.C. and A‐M.X. contributed equally to this work.

## Funding

This study was supported by the National Science and Technology Major Project, 10.13039/501100018537, 2023ZD0506500 and 2024ZD0530000; Jiangsu Province Science and Technology Plan Project, BF2024054; and National Natural Science Foundation of China, 10.13039/501100001809, 82373547 and 82341032.

## Supporting information


**Supporting Information** Additional supporting information can be found online in the Supporting Information section. Table S1.

## Data Availability

Data sharing is not applicable to this article as no datasets were generated or analyzed during the current study.
